# Isolation and Characterization of a Replication-Competent Molecular Clone of an HIV-1 Circulating Recombinant Form (CRF33_01B)

**DOI:** 10.1371/journal.pone.0006666

**Published:** 2009-08-18

**Authors:** Kok Keng Tee, Shigeru Kusagawa, Xiao-Jie Li, Narumi Onogi, Maya Isogai, Saiki Hase, Rie Uenishi, Huanan Liao, Adeeba Kamarulzaman, Yutaka Takebe

**Affiliations:** 1 Laboratory of Molecular Virology and Epidemiology, AIDS Research Center, National Institute of Infectious Diseases, Shinjuku-ku, Tokyo, Japan; 2 Department of Medicine, Faculty of Medicine, University of Malaya, Kuala Lumpur, Malaysia; National AIDS Research Institute, India

## Abstract

A growing number of emerging HIV-1 recombinants classified as circulating recombinant forms (CRFs) have been identified in Southeast Asia in recent years, establishing a molecular diversity of increasing complexity in the region. Here, we constructed a replication-competent HIV-1 clone for CRF33_01B (designated p05MYKL045.1), a newly identified recombinant comprised of CRF01_AE and subtype B. p05MYKL045.1 was reconstituted by cloning of the near full-length HIV-1 sequence from a newly-diagnosed individual presumably infected heterosexually in Kuala Lumpur, Malaysia. The chimeric clone, which contains the 5′ LTR (long terminal repeat) region of p93JP-NH1 (a previously isolated CRF01_AE infectious clone), showed robust viral replication in the human peripheral blood mononuclear cells. This clone demonstrated robust viral propagation and profound syncytium formation in CD4^+^, CXCR4-expressing human glioma NP-2 cells, indicating that p05MYKL045.1 is a CXCR4-using virus. Viral propagation, however, was not detected in various human T cell lines including MT-2, M8166, Sup-T1, H9, Jurkat, Molt-4 and PM1. p05MYKL045.1 appears to proliferate only in restricted host range, suggesting that unknown viral and/or cellular host factors may play a role in viral infectivity and replication in human T cell lines. Availability of a CRF33_01B molecular clone will be useful in facilitating the development of vaccine candidates that match the HIV-1 strains circulating in Southeast Asia.

## Introduction

The ability of the human immunodeficiency virus type 1 (HIV-1) to recombine at high frequency is a critical evolutionary feature that dictates the extensive diversification of HIV-1 [1], [2]. Intersubtype recombination among distinct HIV-1 strains generating various types of circulating recombinant form (CRF) have been documented worldwide [3], [4] in which CRF01_AE and CRF02_AG are two major representative recombinants that are found in Southeast Asia and West Africa, respectively. In the Southeast Asia epidemic, continual recombination events involving CRF01_AE and subtype B' (subtype B variant of Thai origin) have formed new radiations of novel CRFs, including CRF15_01B [5], CRF33_01B [6], and CRF34_01B [7]. Such vigorous expansion of CRFs in this region with 4.0 million HIV-1 infections (as of December 2007) [8] results in epidemiological diversity of growing complexity, a phenomenon that has become increasingly similar to that observed in the African continent [9]. Although CRF01_AE remains the dominant circulating strain, CRF15_01B and CRF33_01B have in recent years been estimated to cause thousands of infections in Thailand and Malaysia, respectively [10]. In order to facilitate the development and validation of neutralization assays for use in vaccine clinical trials in the region, reagents and panels of well characterized HIV isolates based on epidemiologically relevant HIV strains are essential. In this study, we constructed an infectious DNA clone CRF33_01B that is a major circulating strain in Malaysia.

## Materials and Methods

### Virus isolation

EDTA-treated blood specimens were collected from an HIV-infected individual, a 43-year-old heterosexual male attending the HIV clinic of the University Malaya Medical Center in Kuala Lumpur [6]. The patient has given written consent to the use of his blood samples for research and publication. The serostatus of the patient was established by enzyme-linked immunosorbent assay (ELISA) and Western blot analysis in July 2005. The subject was receiving antiretroviral treatment consisting of zidovudine, lamivudine and efavirenz at the point of blood collection in August 2005, with plasma viral load and CD4^+^ cell count recorded at 4300 HIV RNA copies/ml and 121 cells/mm^3^, respectively. Upon sample collection, peripheral blood mononuclear cells (PBMCs) were separated by Ficoll-Hypaque density gradient centrifugation (Amersham Biosciences AB, Uppsala, Sweden) according to manufacturer's protocol. For virus isolation, PBMCs were co-cultured with phytohemagglutinin (PHA) (1 µg/ml)-stimulated CD8^+^ T cell-depleted PBMCs (Miltenyi Biotec GmbH, Bergisch Gladbach, Germany) from HIV-negative healthy donors in RPMI 1640 containing 10% fetal calf serum and interleukin-2 (20 U/ml) for 30 days. Virus production was monitored by the virion-associated reverse transcriptase (RT) assay described previously [11]. HIV-infected PBMCs were harvested and proviral DNA was isolated with guanidine detergent (Invitrogen, Carlsbad, CA).

### Reconstitution of chimeric infectious molecular clone for CRF33_01B

**Figure 1:**
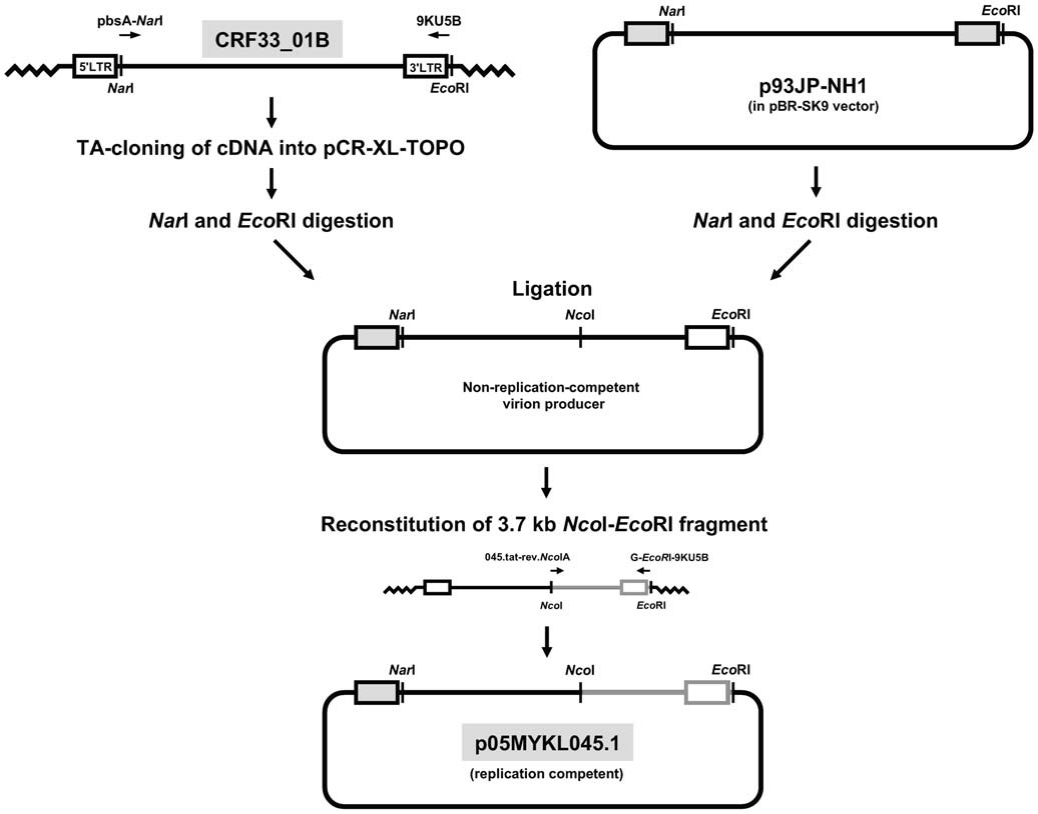
Outline for constructing a replication-competent DNA clone of HIV-1 CRF33_01B (p05MYKL045.1). Near full-length proviral DNA of CRF33_01B was amplified by long-range PCR using pbsA-*Nar*I and 9KU5B primers and TA-cloned into a pCR-XL-TOPO vector. DNA clone containing CRF33_01B genome and p93JP-NH1, an infectious clone of CRF01_AE origin [13], were linearized by *Nar*I and *Eco*RI. The *Nar*I-*Eco*RI fragment from the respective CRF33_01B proviral DNA was directionally ligated with the pBR-SK2 vector that contains the p93JP-NH1 5′ long terminal repeat (LTR) to reconstitute a chimeric full-length construct. Each clone was purified and transformed into HeLa cells to determine proviral replication. Constructs producing non-replicating viruses were then rescued by reconstituting a 3.7 kb proviral fragment (with *Nco*I and *Eco*RI sites) that includes the functional *env* gene to recover an infectious CRF33_01B clone, designated as p05MYKL045.1. Restriction enzyme sites in the DNA and the p93JP-NH1-derived 5′ LTR region in p05MYKL045.1 (shaded) are indicated. Refer text for complete descriptions.

Near full-length proviral DNA was amplified using primers pbsA-*Nar*I (5′-AGT GGC GCC CGA ACA GG-3′; HXB2 634–650) [12] (palindromic sequence recognized by the specified restriction enzyme is underlined) and 9KU5B (5′-GGT CTG AGG GAT CTC TAG TTA CCA G-3′; HXB2 9666–9690) by Expand Long Template PCR System (Roche Diagnostic GmbH, Penzberg, Germany), purified and TA-cloned in the pCR-XL-TOPO vector (Invitrogen, Carlsbad, CA). Positive clones harboring the near full-length insert were screened and selected for subcloning into p93JP-NH1, a previously described replication-competent DNA clone of CRF01_AE strain (in a modified pBR-SK9 vector containing multiple cloning sites) that showed robust replication capacity in various CD4^+^, CXCR4/CCR5-expressing cells [13]. Briefly, *Nar*I and *EcoR*I digestion (convenient and unique restriction enzyme cloning sites in both pCR-XL-TOPO and pBR-SK9) was performed to allow subcloning of near full-length proviral DNA into the background of p93JP-NH1. Corresponding fragments of proviral DNA (insert) and p93JP-NH1 (vector) were unidirectionally ligated to generate a chimeric DNA clone with a 5′ long terminal repeat (LTR) derived from p93JP-NH1 (Figure 1). For plasmid production, plasmid was transformed into the chemically-competent One Shot Stbl3 *E. coli* (Invitrogen, Carlsbad, CA) following the manufacturer's protocol, isolated, and purified for genome-specific sequencing to confirm the presence of the desired insert. To determine plasmid replication, HeLa cells (5×10^4^ cells) in 24-well plate were transfected with 0.4 µg plasmid using FuGENE 6 transfection reagent (Roche Diagnostic GmbH, Penzberg, Germany) and culture supernatant was harvested 2 days post-transfection for detecting the RT activity. Using the RT-positive culture supernatant, growth kinetics of each clone was assessed in CXCR4- or CCR5-expressing CD4^+^ human glioma NP-2 cells [14] to determine: (1) virus infectivity, and (2) coreceptor usage. Briefly, HeLa cells supernatant containing 5×10^5^ counts per minute (cpm) of ^32^P activity per 100 µl medium was filtered (0.45 µm pore size filter) and inoculated into 5×10^3^ NP-2 cells in a 96-well plate. Culture assay was incubated in triplicate for about 3 weeks post-infection and cells were microscopically examined for cytopathic effect (CPE). Viral infectivity was measured by detecting RT production in culture supernatant every 2 days. Other established molecular clones of various coreceptor tropisms were included as positive controls (pNL(AD8), CCR5-tropic [15]; pNL4-3, CXCR4-tropic [16]; p93JP-NH1, dual-tropic), whereas negative control wells were mock-infected with culture medium only.

### Replication kinetics in human PBMCs and T cell lines

To test the replication kinetics and coreceptor tropism, viral stock was prepared by transfecting 5 µg HIV-1 clone into 6×10^5^ HeLa cells using the FuGENE 6 transfection reagent (Roche Diagnostic GmbH, Penzberg, Germany). Culture supernatant was harvested 2 days post-transfection. The production of viral RNA *in situ* was measured by RT assay, and virus containing supernatant was filtered and stored at –80°C until use. To examine the growth kinetics in PBMCs, 100 µl medium containing 2×10^5^ of CD8^+^ T cell-depleted, PHA-activated PBMCs from healthy donors were inoculated with viral stock in a 96-well plate. Virus containing supernatant equivalent to ^32^P activity of 5×10^5^ cpm per 100 µl were used for infection assay. After overnight adsorption, cultures were washed twice and fresh RPMI 1640 medium containing 10% fetal calf serum and interleukin-2 (20 U/ml) was added to a final volume of 200 µl. Each assay was prepared in triplicate. Culture medium was harvested for RT assay and replenished with fresh medium every 3 days until 30 days post-infection. In addition to human PBMCs, viral propagation was tested in CD4^+^ human T cell lines, including MT-2, M8166, Sup-T1, H9, Jurkat, Molt-4 and PM1, and also CEMx174. Similar to PBMCs, 2×10^4^ of these cells were inoculated with virus stock under the same experimental conditions and settings, and virion-associated RT activities were measured every 3 days for 30 days post-infection.

### Phylogenetic and recombination analysis

Near full-length genome of the construct was sequenced using primer walking method described previously [6]. Phylogenetic tree was reconstructed to elucidate the genetic relationship of the construct with its primary isolate, other CRF33_01B, CRF15_01B and CRF33_01B reference sequences. In addition, parental CRF01_AE and subtype B' sequences of Asian origin were retrieved from the HIV Sequence Database [3] and included in the analysis. Nucleotide sequences were aligned and adjusted manually. Phylogenetic tree was plotted using neighbor-joining method implemented in MEGA version 4.0 [17] with 1000 bootstrap replicates, and bootscanning was performed in SimPlot version 3.5 [18] to confirm the recombination structure.

## Results and Discussion

In this article, we reconstituted a chimeric infectious molecular clone of CRF33_01B, which contains the 5′ LTR derived from p93JP-NH1. The experimental outline is depicted in Figure 1. From approximately 100 clones of near full-length proviral DNA generated, only one construct that replicated in HeLa cells (but showed no infectivity in NP-2 cells) was identified, indicating the proportions of replication-competent viral particles are low. To rescue the molecular construct that produces non-infectious virions, the envelope (*env*) gene, which plays a central role in viral attachment, coreceptor usage and infectivity, was replaced with potentially functional *env* gene from patient's chromosomes. Sub-genomic region that spans the *tat*/*rev* gene to the 3′ LTR downstream (approximately 3.7 kb) was amplified from the proviral DNA by Expand Long Template PCR System using 045.tat-rev.*Nco*IA (5′-GGC TTA GGC ATC TCC CAT GG-3′; HXB2 5954–5973) and G-*EcoR*I-9KU5B (5′-GGA ATT C-9KU5B-3′) primers. PCR amplimers were cleaved with *Nco*I and *EcoR*I and reconstituted into the non-replication-competent virion producer (Figure 1). Viral replication kinetics/coreceptor usage was then tested in NP-2 cells, and also in PBMCs. The resultant replication-competent molecular clone was designated as p05MYKL045.1.

**Figure 2:**
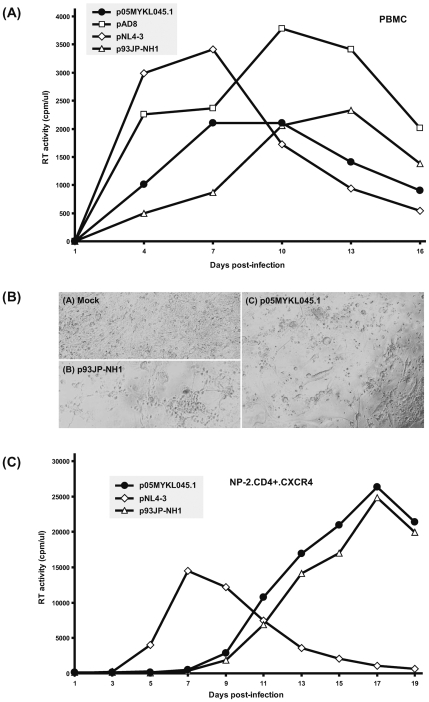
Replication kinetics and virological properties of HIV-1 CRF33_01B molecular clone p05MYKL045.1. (A) To test the replication dynamics of p05MYKL045.1 in human PBMCs, viral stock equivalent to 5×10^5^ cpm of ^32^P activity per 100 µl was inoculated into 2×10^5^ phytohemagglutinin-stimulated CD8^+^ T cell-depleted PBMCs. Assay was prepared in triplicate and cells were incubated for 30 days. RT readout monitored at every 3 days showed peak replication at day 7 and 10 post-infection, with ^32^P activity of about 2100 cpm/µl of medium. Molecular clones of various coreceptor usages were used as positive controls (pNL(AD8), CCR5-tropic; pNL4–3, CXCR4-tropic; p93JP-NH1, dual-tropic). (B) Phase-contrast microscopy of NP-2.CD4.CXCR4 cells infected with p05MYKL045.1 molecular clone. NP-2 cells morphology was examined every 2 days post-infection. Shown here is day 15 post-infection where profound cytopathic effect (CPE), as evident by the presence of multinuclear giant cells (syncytium formation) and cell lyses, was observed in NP-2.CD4.CXCR4 cells infected with p05MYKL045.1 (panel C). Similar CPEs were also noted in control wells infected with p93JP-NH1 (panel B). Negative control (panel A) shows NP-2.CD4.CXCR4 cells mock-infected with virus-free culture medium. (C) Virus propagation and co-receptor usage of p05MYKL045.1 in human glioma NP-2.CD4.CXCR4 cells. Viral inoculum of 5×10^5^ cpm of ^32^P activity per 100 µl medium was used to infect 5×10^3^ NP-2.CD4.CXCR4 cells in triplicate in 96-well plates. Virion-associated RT production was measured at the indicated time points. Peak viral replication of more than 25000 cpm/µl of ^32^P activity was observed at day 17 post-infection.

Replication kinetics of p05MYKL045.1 in CD8^+^ T cell-depleted, PHA-stimulated human PBMCs showed that the peak virus replication was achieved around day 7 and 10 post-infection (Figure 2A). Similar viral propagations in PBMCs were also observed for p93JP-NH1 and other control viruses. To define the coreceptor tropism for p05MYKL045.1, viral replication was tested in CXCR4- or CCR5-expressing CD4^+^ NP-2 cells. Microscopic examinations revealed syncytium formation in p05MYKL045.1-infected NP-2.CD4.CXCR4 cells (Figure 2B), but not in NP-2.CD4.CCR5 cells. Accordingly, virion-associated RT activity was detected in the culture supernatant of NP-2.CD4.CXCR4, but not in NP-2.CD4.CCR5 cells. The peak viral replication was detected at day 17 post-infection (more than 25000 cpm/µl of ^32^P activity) in NP-2.CD4.CXCR4 (Figure 2C). The results also indicated that the peak viral replication for p05MYKL045.1 was about 2-fold higher than that of the commonly used molecular clone pNL4–3, suggesting that p05MYKL045.1 was highly replicative in CXCR4-expressing cell line. Together, our studies demonstrated that p05MYKL045.1 is a CXCR4-using infectious clone that replicates competently in PBMCs and human glioma NP-2 cells.

Next, we tested the replicative capacity of p05MYKL045.1 in various immortalized human CD4^+^ T cell lines (MT-2, M8166, Sup-T1, H9, Jurkat, Molt-4 and PM1) and also CEMx174. No RT production (or profound CPE) was detected for p05MYKL045.1 virus in all cell lines (including MT-2, which is an indicator cell line for CXCR4 tropism [19]) within 30 days post-infection. The data suggest that p05MYKL045.1 virus can only infect and replicate in restricted host range. Such limitations could be due to the specific viral properties in this clone that cannot support its propagation in human T cell lines, despite the fact that the virus replicate well in PBMCs. We note that such observation is not uncommon among other HIV-1 DNA constructs. Yet undefined viral genetic factors and/or complex interaction between viral and host factors may be involved in this phenomenon. This includes the possibility of p05MYKL045.1 virus replicating at very low levels that rendered the RT activity undetectable by our assay, or the relatively low level of CD4 and coreceptors expression in these T cell lines compared to the engineered NP-2 cells that may potentially limit p05MYKL045.1 viral infectivity [13].

**Figure 3:**
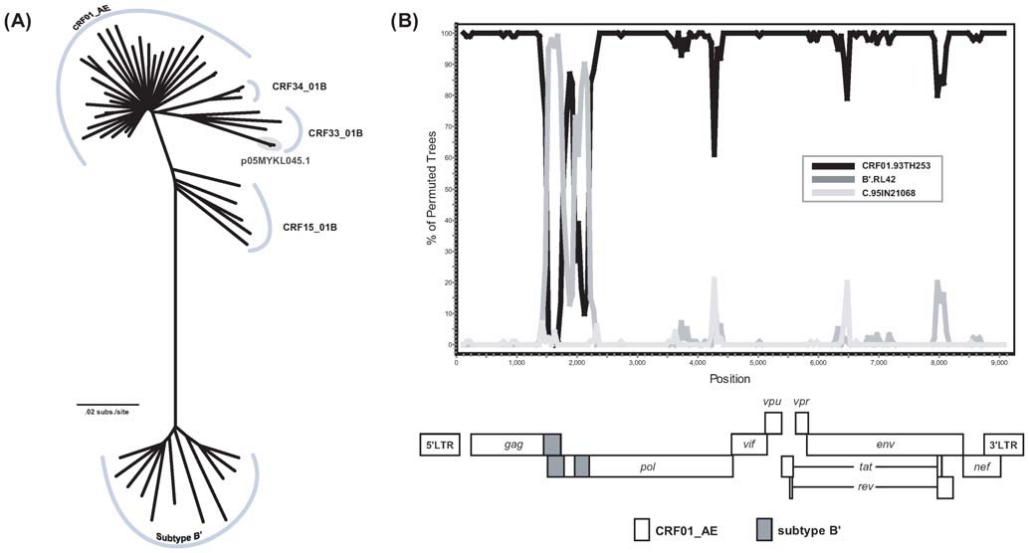
Phylogenetic and recombination analysis of HIV-1 CRF33_01B molecular clone p05MYKL045.1. (A) Phylogenetic reconstructions of HIV-1 CRF01_AE, subtype B', CRF33_01B and other CRF01_AE/B' recombinants of Southeast Asian origin. Near full-length nucleotide sequences were aligned and adjusted manually. Hypervariable and ambiguous sequences along the genome that could not be aligned were stripped. Cladogram was plotted using neighbor-joining method implemented in MEGA version 4.0 [17] with 1000 bootstrap replicates. Reference sequences were retrieved from the HIV Sequence Database [3]. The p05MYKL045.1 genome, determined by primer walking method was clustered closely with its primary parental isolate (shaded). (B) Bootscan plot depicting the recombination structure of p05MYKL045.1 genome. Bootscanning analysis was performed using SimPlot version 3.5 [18] with a sliding window of 250 nucleotides overlapping by 50 nucleotides step size. HIV-1 CRF01.93TH253 and B'.RL42 references were used as the putative parental strains, with C.95IN21068 sequence as the outlier.

The CRF33_01B genome in p05MYKL045.1 construct was 9153 bp in size, spanning all structural and regulatory genes with intact open reading frames. Major insertions or deletions and premature protein terminations were not present across the genome. Phylogenetic reconstructions of the near full-length genomes revealed that p05MYKL045.1 clustered closely with the primary isolate (GenBank accession no. DQ366662), confirming its parental origin (Figure 3A). Bootscan plot also showed that the recombinant structure of p05MYKL045.1 was consistent with other CRF33_01B references, in which two short fragments of subtype B' were present in the *gag* and *pol* regions while the rest of the genome was closely-related to CRF01_AE (Figure 3B) [6]. The nucleotide sequence of p05MYKL045.1 reported in this paper has been deposited in the GenBank database under accession number GQ277610.

Availability of the replication-competent molecular clones that express functional viral gene products enables systematic virological assessment of HIV-1 subtypes/CRFs *in vitro*. Phenotypic and genotypic studies that involve artificial manipulation of viral genomes at DNA level provide significant insights on viral replication, pathogeneses and other biological characteristics. Despite being one of the major sites for CRF01_AE and subtype B-based vaccine efficacy trials, the limited collection of molecular clones of Southeast Asian origin [13], [20]–[22] may present a daunting challenge in developing vaccine candidates that match the locally prevalent and diverse HIV strains especially when there is a lack of standardized reagents for HIV neutralization assays in clinical evaluations. As an emerging, widely distributed CRF in the Southeast Asia region, an CRF33_01B infectious DNA clone is a valuable tool in facilitating the development of broadly effective vaccine candidates.
